# Quantitative Study on Human Error in Emergency Activities of Road Transportation Leakage Accidents of Hazardous Chemicals

**DOI:** 10.3390/ijerph192214662

**Published:** 2022-11-08

**Authors:** Wei Jiang, Zhishun Huang, Zonghao Wu, Huiyuan Su, Xiangping Zhou

**Affiliations:** 1 School of Emergency Management and Safety Engineering, China University of Mining & Technology (Beijing), Ding No. 11 Xueyuan Road, Haidian District, Beijing 100083, China; 2 Beijing Institute of Mechanical and Electrical Engineering, Courtyard 40, Yungang Beili, Fengtai District, Beijing 100074, China

**Keywords:** hazardous chemical, road transport leakage accident, emergency activities, CREAM, human error

## Abstract

The emergency rescue process of road transportation leakage accidents involving hazardous chemicals is complex and includes various emergency activities. A quantitative study of human errors in emergency activities is conducive to seeking the focus of the emergency rescue process. To quantitatively analyze human error in emergency activities during the emergency rescue process of road transportation leakage accidents of hazardous chemicals, sequentially timed events plotting (STEP) and the cognitive reliability and error analysis method (CREAM), were used. First, STEP was used to analyze six laws, regulations and standards, as well as 54 accident cases, to derive 24 emergency activities in the emergency rescue process. Then, CREAM was used to analyze and obtain the probability of human error for each emergency activity. Two high error level emergency activities, five medium error level emergency activities, and seventeen low error level emergency activities were identified after the human error levels of the emergency activities were classified. The results show that two emergency activities, the initial handling of the accident, and cleanup of the leakage site, should be prioritized in the emergency rescue process of road transportation leakage accidents of hazardous chemicals.

## 1. Introduction

Leakage accidents account for the highest proportion of accidents in the process of the road transportation of hazardous chemicals [1], and they frequently result in serious accident outcomes, such as poisoning and asphyxiation, fire and explosion. The effect of emergency rescue in hazardous chemical leakage accidents can directly affect the severity of the consequences of accidents [2].

Regarding the emergency rescue of road transportation leakage accidents of hazardous chemicals, some scholars have studied the emergency process [3,4,5], emergency supervision [6], risk assessment [7] and management [8], as well as other perspectives. Additionally, some scholars have studied specific cases and established emergency rescue frameworks [9], platform systems [10], and so on. However, we found no research on the emergency activities of road transportation leakage accidents of hazardous chemicals. The emergency rescue process consists of corresponding emergency activities, and the correctness and rationality of the emergency activities in the rescue process can effectively guarantee the proper disposal of hazardous chemicals from road transportation leakage accidents [11]. The emergency activities mentioned in this paper refer to activities involved in the emergency rescue process to stop the disaster from getting worse, and secondary accidents from happening, as well as reducing accident risks after the occurrence of a road transportation accident of hazardous chemicals. Human error in emergency activities is a very significant component, and once it happens it often leads to secondary accidents or risks. Therefore, limiting human error in emergency activities can aid in accident control and financial loss reduction throughout the emergency rescue process following a leakage accident of hazardous chemicals.

Based on the above reasons, this paper only focuses on human errors in the emergency rescue process after the accident, and takes the road transport leakage accident of hazardous chemicals as an example for study.

## 2. Materials and Methods

### 2.1. Cognitive Reliability and Error Analysis Method (CREAM)

Regarding the study of human errors, there are the reason model (Swiss Cheese model) [12], human factors analysis and classification system (HFACS) [13], and 24Model [14], among others. However, these models can only qualitatively analyze human errors in emergency activities and cannot quantitatively calculate the probability of human error in emergency activities. CREAM was proposed by Hollnagel, E. in 1998 and contains both qualitative analysis and quantitative analysis [15]. The qualitative analysis function focuses on the retrospective analysis of the accident, which is to trace the observable and unobservable errors that led to the accident through the accident results. The quantitative analysis function examines the likelihood of errors in the execution of the corresponding task steps by personnel in a given activity.

CREAM has been applied in the aviation, nuclear power, and navigation fields [16,17]. The qualitative analysis in CREAM is mainly used in accident analysis [18,19], in which the probability of human error can be analyzed and predicted for emergency activities in the emergency rescue process of road transportation accidents involving hazardous chemicals. In terms of the quantitative analysis of CREAM, some scholars have combined CREAM with other methods to calculate the probability of human error. For example, Marseguerra, M. et al. considered how CREAM could be applied, and suggested calculating the probability of action failure in accordance with how performance conditions would affect the process [20]. To rate performance conditions and estimate the probability of process failure, Felice, F.D. et al. proposed a mixed model of human error probability analysis of CREAM and the systematic human error reduction and prediction approach (SHERPA) to comprehend human behavior, predicted error probability, and used it in the prevention stage of accidents to mitigate damage [21]. Chai, S. et al. modified the common performance conditions (CPC) factors in the original CREAM to improve the applicability of the model in the field of offshore oil and gas production operations [22].

We used the quantitative analysis function in CREAM to determine cognitive activities, error modes, error probabilities and weighting factors [16], and then calculated the human error probabilities of each emergency activity in the emergency rescue process, to derive the focus in the emergency rescue process of road transport leakage accidents of hazardous chemicals.

The sequentially timed events plotting (STEP) method could solve this issue since CREAM is unable to obtain the emergency activities involved in the emergency rescue process of road transportation leakage accidents of hazardous chemicals.

### 2.2. Sequentially Timed Events Plotting (STEP)

STEP, proposed by Benner, S. and Hendrick, S. in 1987, is an accident investigation method that draws and restores the scene of the accident based on factors such as event time development, personnel roles, and corresponding actions [23]. This method, improved by the Norwegian Institute of Technology, focuses on the correlation between people and events and provides a comprehensive process framework for accident investigation and analysis, from the description of the accident process to the identification of safety issues, and then to the formulation of safety recommendations [24].

Research on STEP has focused on two aspects. One is the application of the method to analyze a specific accident type and restore the logical sequence of events that developed over time [25,26]. The other is a comparative study of the differences between STEP and other accident investigation methods [27,28]. The benefits of using the STEP for accident analysis in this paper are that it can clearly show the relationship between time, events, and personnel, it can restore the emergency scenario of a leakage accident, and it can quickly find and extract the emergency activities involved in the emergency rescue process of a road transportation leakage accident of hazardous chemicals.

### 2.3. Combination of STEP and the CREAM

**Figure 1:**
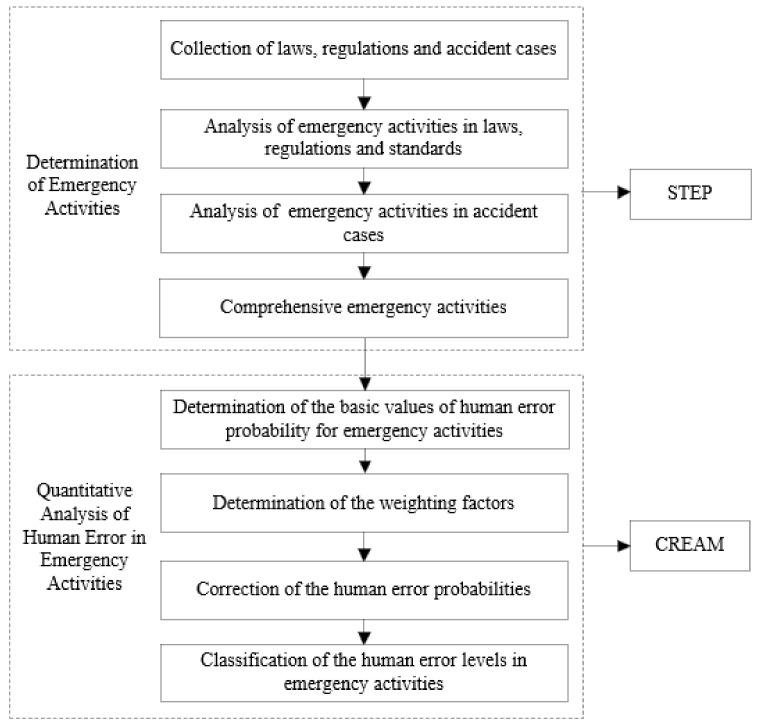
Combination of STEP and CREAM.

STEP and the CREAM were combined in this paper. First, to determine all of the emergency activities included in the emergency rescue process of road transportation leakage accidents of hazardous chemicals, the collected laws, regulations and standards, as well as the emergency rescue process of the accident cases, were analyzed using STEP. Then, CREAM was applied to analyze and calculate the probability of human error in each emergency activity. Finally, according to the probability of human error of emergency activities, the error level was divided to seek the focus of the emergency rescue process of the road transportation leakage accidents of hazardous chemicals. The combination of STEP and CREAM is shown in Figure 1. 

## 3. Analysis of Emergency Activities

To guarantee the comprehensiveness of the analysis, the analysis of the emergency activities of road transportation leakage accidents of hazardous chemicals in this paper included three aspects. The first was to analyze the emergency activities involved in the emergency rescue process stipulated by laws, regulations and standards; the second was to analyze the emergency activities involved in the road transportation leakage accidents of hazardous chemicals, and the third was to synthesize the two aspects to obtain all emergency activities involved in the emergency rescue process.

### 3.1. Analysis of Emergency Activities in Laws, Regulations and Standards

A total of six laws, regulations and standards related to the transportation leakage accidents of hazardous chemicals were collected from the websites of the Ministry of Emergency Management of the People’s Republic of China [29], the China Chemical Safety Association [30] and the Ministry of Emergency Management Chemical Registration Center [31], as shown in Table 1.

According to the “Emergency Response Law of the People’s Republic of China” (President’s Decree No. 69), the emergency rescue process is divided into four stages: prevention, preparation, response, and recovery, in which the prevention stage and preparation stage are measures taken before the occurrence of an accident. These two stages were not addressed in this paper because the research focused on emergency activities following an accident. The response stage refers to the stage of carrying out the emergency disposal and rescue work during the emergency rescue process, which can be divided into response after receiving the accident information and emergency disposal of the accident site in accordance with chronological order. Therefore, the response stage was further divided into the initial response stage and on-site disposal stage for a detailed description of the emergency rescue process. The accident recovery stage includes short-term recovery and long-term recovery. This paper focused on the short-term recovery of the emergency activities taken after the accident, which was redefined as the aftermath disposal stage to avoid ambiguity.

In summary, we divided the emergency rescue process of road transportation leakage accidents of hazardous chemicals into three stages: initial response, on-site disposal and aftermath disposal. According to the laws, regulations and standards in Table 1, the emergency activities corresponding to each stage were identified, and the specific basis of identification and emergency activities are shown in Table 2.

**Figure 2:**
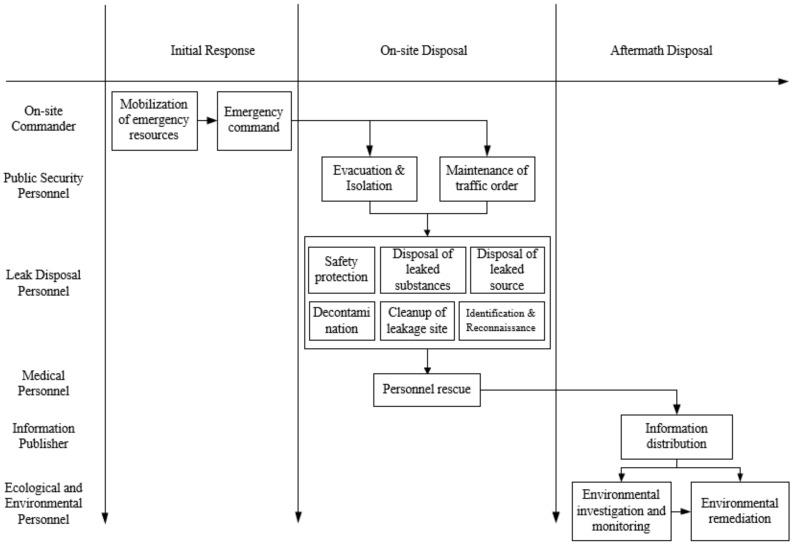
Emergency activities: laws, regulations and standards.

The emergency activities were mapped by STEP according to all of the emergency activities in Table 2, as shown in Figure 2.

### 3.2. Analysis of Emergency Activities in Accident Cases

Considering that the actual emergency rescue process is complicated, and more emergency activities exist than those in laws, regulations and standards after the accident, to ensure the comprehensive analysis of emergency activities, 54 cases of road transportation leakage accidents of hazardous chemicals from January 2019 to January 2022 were collected from the Ministry of Emergency Management of the People’s Republic of China [29], the China Chemical Safety Association [30] and the Ministry of Emergency Management Chemical Registration Center [31], and were analyzed by STEP according to the 54 accident investigation reports.

**Figure 3:**
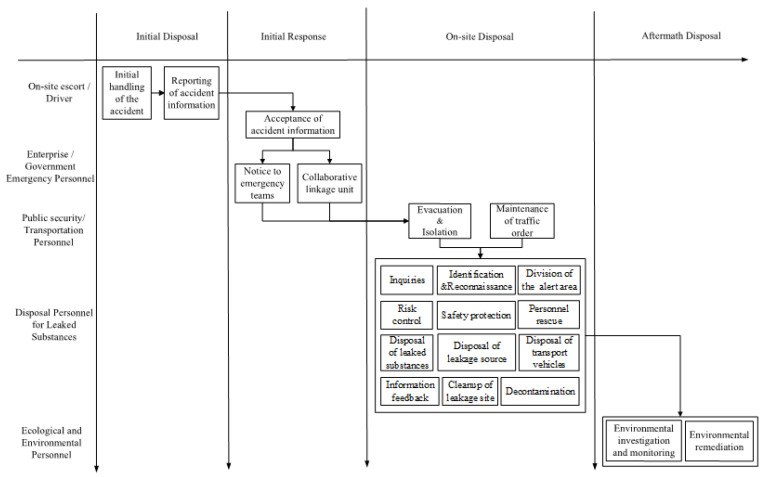
Emergency activities in 54 accident cases.

We added the initial disposal of accidents to the three stages in Table 2: initial response, on-site disposal, and aftermath disposal. The reason was that after the accident, the personnel at the accident site carry out simple initial on-site disposal according to the accident situation before the rescue personnel arrive. The emergency activities were mapped by STEP according to 54 accident investigation reports, as shown in Figure 3.

### 3.3. Summary of Analysis Results

**Figure 4:**
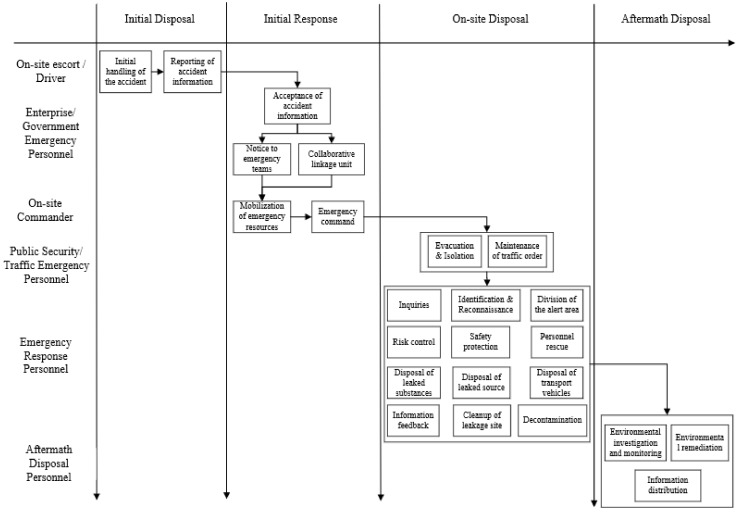
Emergency activities map of road transportation leakage accidents of hazardous chemicals.

Combining Figure 2 and Figure 3, emergency activities included in the emergency rescue process of road transportation leakage accidents of hazardous chemicals can be obtained, as shown in Figure 4.

According to Figure 4, 24 emergency activities for road transportation leakage accidents of hazardous chemicals were obtained, as shown in Table 3.

## 4. Quantitative Analysis of Human Errors for Emergency Activities

### 4.1. Analysis of the Probability of Human Error for Emergency Activities

The probability of human error during emergency activities was studied using CREAM. First, cognitive activities in CREAM corresponding to the emergency activities in this paper were established. The associated error modes were identified in accordance with the appropriate cognitive activities, after which the basic values of the probability of human error were established. Then, weighting factors were determined according to the corresponding failure modes. Finally, the probability of human error of emergency activities was determined by correcting the basic values of the probability of human error of emergency activities using a weighting factor. The selection of the basic values of the probability of human error, the calculation of the weighting factor, and the error modes listed in Table 4 were all based on the literature [15].

#### 4.1.1. Basic Values of the Probability of Human Error for Emergency Activities

To determine the basic value of the probability of human error during emergency activities, we first determined what cognitive activities in CREAM corresponded to emergency activities, then determined the error modes of emergency activities according to the cognitive activities, and finally obtained the basic value of the probability of human error of emergency activities through the probability corresponding to the error mode provided by CREAM.

According to the cognitive activities provided by CREAM, the specific emergency activities in Table 4 were categorized as corresponding cognitive activities. The error modes of emergency activities were determined using the laws, regulations, and standards in Table 1, and 54 accident case descriptions in conjunction with the cognitive activities offered by CREAM, as shown in Table 4. The cognitive activities in Table 4 are the common results extracted from the 54 accident cases; some cognitive activities were removed and not reflected in Table 4 because of their low occurrence frequency in the 54 cases. The number settings, meanings and values of e1, e2, e3, e4, e5, o1, o2, o3, i1, i2, i3, p1 and p2 in Table 4 and Table 5 were from the literature [15].

The error probability corresponding to cognitive activities and error modes was provided in CREAM. After determining the cognitive activities corresponding to emergency activities and the corresponding error modes, the probability of error corresponding to the error modes was obtained according to the literature [15] and was used as the basic values of analysis, as shown in Table 5.

#### 4.1.2. Determining Weighting Factors

The basic values of the probability of human error for 24 emergency activities are shown in Table 5, on the basis of which we further determined the weighting factors corresponding to the probability of human error for emergency activities.

For the determination of the probability of human error weighting factors, the steps are as follows.

(1) The evaluation levels of 24 emergency activities were evaluated based on the descriptions of the corresponding emergency activities in 54 road transportation leakage accidents of hazardous chemicals. The evaluation items and the corresponding evaluation levels are shown in Table 6.
(2) The weighting factors attributed to each emergency activity were determined. Based on Table 4, the error mode of each emergency activity was obtained, and the weighting factors of the emergency activities for the nine evaluation items were determined based on the evaluation level of the previous step.
(3) The average weighting factors for each emergency activity were determined. After the weighting factors were determined by evaluating the nine items in turn, the average of the nine items was obtained, which was the final probability of human error weighting factor for the emergency activities.

Regarding the determination of the weighting factors, CREAM uses subjective methods such as expert evaluation and self-assumptions. We analyzed the descriptions of nine items in 54 accident cases and determined them strictly according to the Guide for command of emergency rescue in hazardous chemical accidents (AQ-T3052-2015) on the emergency rescue process. For example, the rescue process of an accident has a reasonable organizational structure, timely response from all departments, and perfect coordination and preparation, so the level of adequacy of organizational of this accident were evaluated as very efficient, and similar accident cases were given the same evaluation, otherwise other evaluation levels were considered. In addition, the evaluation results of 54 accident cases were taken as the mean value to further increase the objectivity of the study.

Taking the evaluation of “adequacy of organization” in the first emergency activity (the initial handling of the accident) as an example, the specific application was as follows:

(1) From descriptions of the adequacy of organization in emergency activities of 54 road transportation leakage accidents of hazardous chemicals, 16 out of 54 accident cases were very efficient, 30 were efficient and 8 were inefficient. Taking their average, the evaluation level of the emergency activity of the initial handling of the accident was determined to be efficient. The remaining eight evaluation items in Table 7 were the same.
(2) According to Table 4, the human error mode of the initial handling of the accident was execution (e). According to the evaluation level of “Adequacy of organization” determined in Table 7, the weighting factor of the initial handling of the accident for “Adequacy of organization” was 1.0. The weighting factors of the remaining eight evaluation items in Table 7 were determined in the same manner.
(3) According to step (2), the weighting factors of the initial handling of the accident for the nine evaluations were obtained, and the nine weighting factors were averaged to obtain the average weighting factor of the initial handling of the accident as an emergency activity, which was 1.09.

According to the above steps, the weighting factors of the remaining 23 emergency activities were determined, as shown in Table 8.

After the basic values and the average weighting factors of the probability of human error for emergency activities were determined, the adjusted values of the probability of human error were determined.

#### 4.1.3. Probability of Human Error Adjustment Values for Emergency Activities

The adjusted values of the probability of human error of emergency activities were determined based on the basic values of the probability of human error (Table 5) and the average weighting factors (Table 8). By multiplying the basic values of the probability of human error and the weighting factors, the adjusted value of the probability of human error of emergency activities was obtained, as shown in Table 9.

## 5. Results

The probability of human error for the emergency activities analyzed in Table 9 is summarized as follows:

The error probability of the highest probability of human error is 3.27% for the initial handling of the accident and the cleanup of the leakage site.

The probability of human error is 1.06% for all three emergency activities: collaborative linkage unit, mobilization of emergency resources and emergency command.

The probability of human error is 0.77% for the two emergency activities: identification and reconnaissance and environmental investigation and monitoring.

The probability of human error is 0.33% for the 14 emergency activities: reporting of accident information, notice to emergency teams, maintenance of traffic order, inquiries, risk control, safety protection, personnel rescue, disposal of leaked substances, disposal of leaked source, disposal of transport vehicles, information feedback, decontamination, environmental remediation and information distribution.

The error probability of the lowest probability of human error is 0.05% for the acceptance of accident information, evacuation and isolation and division of the alert area.

## 6. Discussion

To make our results clearer and easier to judge, we developed a human error ranking for 24 emergency activities based on probability differences.

Based on the probability ranking of each emergency activity, the difference between adjacent probabilities was analyzed, and it was found that the maximum probability difference between the emergency activities was 2.21%, followed by 0.44%. According to the difference between the probabilities, the results of the probability of human error analysis of the 24 emergency activities were classified as high (X ≥ 3%), medium (0.5% ≤ X < 3%), and low (0 ≤ X < 0.5%), as shown in Table 10.

From Table 10, there were two high error level emergency activities, namely, initial handling of the accident and cleanup of leakage site; five medium error level emergency activities, namely, collaborative linkage unit, mobilization of emergency resources, emergency command, collaborative linkage unit, mobilization of emergency resources and emergency command; and seventeen low error level emergency activities. 

The reason for the high probability of human error in initial handling of the accident is that at the beginning stage of the accident the site personnel are affected by their psychological and physiological state, and it is easy to make human errors that lead to improper disposal of the accident, affecting the accident emergency rescue process. As for cleanup when the leakage site in the accident disposal is near the end of the rescue, improper cleaning may cause serious secondary accidents due to inattention. In addition, when these two emergency activities related to disposal at the accident site, the process is susceptible to human error due to the impact on the accident site and the surrounding environment.

In the emergency rescue process of road transportation leakage accidents involving hazardous chemicals, the initial handling of the accident and cleanup of the leakage site should be given more attention.

## 7. Conclusions

We used STEP and the CREAM to quantitatively analyze human errors in the emergency rescue process of road transportation leakage accidents of hazardous chemicals and reached the following conclusions.

Twenty-four emergency activities in the emergency rescue process of road transportation leakage accidents of hazardous chemicals were considered. The probabilities of human error of 24 emergency activities were obtained, and three types of human error levels were classified.

In the emergency rescue process of road transportation leakage accidents of hazardous chemicals, focusing on and reducing human errors in the two emergency activities of the initial handling of the accident and cleanup of the leakage site can help the emergency rescue to be more effective.

In future research, we will continue to collect more information to further classify the emergency activities, which will help to enhance the comprehensiveness as well as the accuracy of human error level classification of emergency activities.

## Figures and Tables

**Table 1:** Laws, regulations and standards.

No.	Name of the Laws, Regulations and Standards	Nature
1	“Emergency response law of the people’s republic of China” (President’s Decree No. 69)	Law
2	“Rules of transportation, loading and unloading of dangerous goods by automobile” (JT618-2004)	Department regulation
3	“The regulation of automobile transportation of dangerous goods” (JT617-2004)	Department regulations
4	“Requirements on emergency materials equipment for hazardous chemical enterprises” (GB 30077-2013)	National standard
5	“Guide for command of emergency rescue in hazardous chemical accidents” (AQ-T3052-2015)	Industry standard
6	“Guide for disposal of hazardous chemical leakage accident” (GA/T 970-2011)	Industry standard

**Table 2:** Emergency activities in laws, regulations and standards.

Stage	Persons Involved	Emergency Activities	Contents of Laws, Regulations and Standards
Initial response	On-site commander	Mobilization of emergency resources	Command and arrange emergency rescue personnel; allocate resources according to the accident situation.
		Emergency command	Formulate a scientific and reasonable rescue plan and conduct unified command and implementation.
On-site disposal	Public security personnel	Evacuation& Isolation	Set warning signs at the boundary of the warning and isolation area; assign special personnel to be responsible for warning
		Maintenance oftraffic order	The road leading to the scene of the accident shall be subject to traffic control, and irrelevant vehicles are strictly forbidden to enter; clear the main traffic roads to ensure the smoothness of the roads
	Leak disposal personnel	Safety protection	Take effective measures to protect yourself according to the hazard characteristics of hazardous chemicals
		Disposal of leaked substances	Leakage control should be carried out simultaneously with leakage source control
		Disposal of leaked source	
		Decontamination	Set up decontamination stations at the junction of hazardous and safe areas
		Cleanup of leakage site	Thoroughly remove the residual toxic and hazardous gases from all parts of the accident site
		Identification & Reconnaissance	Dynamically monitor the concentration and diffusion of combustible, toxic and hazardous chemicals
	Medical personnel	Personnel rescue	Carry life-saving equipment to quickly enter the scene; distressed people in danger will be transferred to a safe area
Aftermath disposal	Information publisher	Information distribution	Information distribution should be timely, accurate, objective and comprehensive
	Ecological and environmental personnel	Environmental investigation and monitoring	Environmental protection departments are responsible for environmental monitoring and supervision, coordination and participation in the emergency disposal of environmental pollution
		Environmental remediation	Environmental protection departments are responsible for environmental pollution tracking and monitoring, guiding the post-disaster environmental recovery work

**Table 3:** Emergency activities of road transportation leakage accidents of hazardous chemicals.

No.	Emergency Activities	No.	Emergency Activities	No.	Emergency Activities
1	Initial handling of the accident	9	Maintenance of traffic order	17	Disposal of leaked source
2	Reporting of accident information	10	Inquiries	18	Disposal of transport vehicles
3	Acceptance of accident information	11	Identification & Reconnaissance	19	Information feedback
4	Notice to emergency teams	12	Division of the alert area	20	Cleanup of leakage site
5	Collaborative linkage unit	13	Risk control	21	Decontamination
6	Mobilization of emergency resources	14	Safety protection	22	Environmental investigation and monitoring
7	Emergency command	15	Personnel rescue	23	Environmental remediation
8	Evacuation and Isolation	16	Disposal of leaked substances	24	Information distribution

**Table 4:** Emergency activities, cognitive activities and error modes.

No.	Emergency Activities	Cognitive Activities	Error Modes			
			Observation(o)	Interpretation (i)	Planning (p)	Execution (e)
1	Initial handling of the accident	Execution				e5
2	Reporting of accident information	Communication				e2
3	Acceptance of accident information	Record				e3
4	Notice to emergency teams	Communication				e2
5	Collaborative linkage unit	Co-ordinate		i2		
6	Mobilization of emergency resources	Planning		i2		
7	Emergency command	Planning		i2		
8	Evacuation& Isolation	Execution				e3
9	Maintenance of traffic order	Maintain				e1
10	Inquiries	Communication				
11	Identification & Reconnaissance	Observation	o2			
12	Division of the alert area	Execution				
13	Risk control	Execution				e1
14	Safety protection	Execution				e1
15	Personnel rescue	Execution				e2
16	Disposal of leaked substances	Execution				e1
17	Disposal of leaked source	Execution				e1
18	Disposal of transport vehicles	Execution				e1
19	Information feedback	Communication				e2
20	Cleanup of leakage site	Execution				e5
21	Decontamination	Execution				e1
22	Environmental investigation and monitoring	Monitor	o3			
23	Environmental remediation	Execution				e1
24	Information distribution	Execution				e2

**Table 5:** Basic values of human error probability for emergency activities.

No.	Emergency Activities	Error Modes	Error Probability
1	Initial handling of the accident	Action missed (e5)	0.03
2	Reporting of accident information	Action performed at wrong time (e2)	0.003
3	Acceptance of accident information	Action on wrong object (e3)	0.0005
4	Notice to emergency teams	Action performed at wrong time (e2)	0.003
5	Collaborative linkage unit	Decision error (i2)	0.01
6	Mobilization of emergency resources	Decision error (i2)	0.01
7	Emergency command	Decision error (i2)	0.01
8	Evacuation & Isolation	Action on wrong object (e3)	0.0005
9	Maintenance of traffic order	Execution of wrong type performed (e1)	0.003
10	Inquiries	Action performed at wrong time (e2)	0.003
11	Identification & Reconnaissance	Wrong identification made (o2)	0.007
12	Division of the alert area	Action on wrong object (e3)	0.0005
13	Risk control	Execution of wrong type performed (e1)	0.003
14	Safety protection	Execution of wrong type performed (e1)	0.003
15	Personnel rescue	Execution of wrong type performed (e1)	0.003
16	Disposal of leaked substances	Execution of wrong type performed (e1)	0.003
17	Disposal of leaked source	Execution of wrong type performed (e1)	0.003
18	Disposal of transport vehicles	Execution of wrong type performed (e1)	0.003
19	Information feedback	Action performed at wrong time (e2)	0.003
20	Cleanup of leakage site	Action missed (e5)	0.03
21	Decontamination	Execution of wrong type performed (e1)	0.003
22	Environmental investigation and monitoring	Observation not made (o3)	0.007
23	Environmental remediation	Execution of wrong type performed (e1)	0.003
24	Information distribution	Action performed at wrong time (e2)	0.003

**Table 6:** Weighting factors.

Evaluation Items	Evaluation Level	Error Mode Weights			
		o	i	p	e
Adequacy of organization	Very efficient	1.0	1.0	0.8	0.8
	Efficient	1.0	1.0	1.0	1.0
	Inefficient	1.0	1.0	1.2	1.2
	Deficient	1.0	1.0	2.0	2.0
Working conditions	Advantageous	0.8	0.8	1.0	0.8
	Compatible	1.0	1.0	1.0	1.0
	Incompatible	2.0	2.0	1.0	2.0
Adequacy of man-Machine Interface and operational support	Supportive	0.5	1.0	1.0	0.5
	Adequate	1.0	1.0	1.0	1.0
	Tolerate	1.0	1.0	1.0	1.0
	Inappropriate	5.0	1.0	1.0	5.0
Availability of Pre-planning	Appropriate	0.8	1.0	0.5	0.8
	Acceptable	1.0	1.0	1.0	1.0
	Inappropriate	2.0	1.0	5.0	2.0
Feature of emergency goals	Fewer than capacity	1.0	1.0	1.0	1.0
	Matching current capacity	1.0	1.0	1.0	1.0
	More than capacity	2.0	2.0	5.0	2.0
Available time	Adequate	0.5	0.5	0.5	0.5
	Temporarily capacity	1.0	1.0	1.0	1.0
	Continuously capacity	5.0	5.0	5.0	5.0
Operation time	Day-time	1.0	1.0	1.0	1.0
	Night-time	1.2	1.2	1.2	1.2
Adequacy of training and experience of emergency personnel	Adequate, high experience	0.8	0.5	0.5	0.8
	Adequate, limited experience	1.0	1.0	1.0	1.0
	Inadequate	2.0	5.0	5.0	2.0
Crew collaboration quality	Very efficient	0.5	0.5	0.5	0.5
	Efficient	1.0	1.0	1.0	1.0
	Inefficient	10	1.0	1.0	1.0
	Deficient	2.0	2.0	2.0	5.0

**Table 7:** Determination of weighting factor for initial handling of accidents.

Evaluation Items	Evaluation Level	Error Modes Weights
Adequacy of organization	Efficient	1.0
Working conditions	Incompatible	2.0
Adequacy of man-Machine Interface and operational support	Tolerate	1.0
Availability of Pre-planning	Acceptable	1.0
Feature of emergency goals	More than capacity	2.0
Available time	Adequate	0.5
Operation time	Day-time	1.0
Adequacy of training and experience of emergency personnel	Adequate, high experience	0.8
Crew collaboration quality	Very efficient	0.5

**Table 8:** Twenty-four emergency activity weighting factors.

No.	Emergency Activities	Error Modes	Weighting Factors	No.	Emergency Activities	Error Modes	Weighting Factors
1	Initial handling of the accident	e5	1.09	13	Risk control	e1	1.09
2	Reporting of accident	e2	1.09	14	Safety protection	e1	1.09
3	Acceptance of accident information	e3	1.09	15	Personnel rescue	e1	1.09
4	Notice to emergency teams	e2	1.09	16	Disposal of leaked substances	e1	1.09
5	Collaborative linkage unit	i2	1.06	17	Disposal of leaked source	e1	1.09
6	Mobilization of emergency resources	i2	1.06	18	Disposal of transport vehicles	e1	1.09
7	Emergency command	i2	1.06	19	Information feedback	e2	1.09
8	Evacuation & Isolation	e3	1.09	20	Cleanup of leakage site	e5	1.09
9	Maintenance of traffic order	e1	1.09	21	Decontamination	e1	1.09
10	Inquiries	e2	1.09	22	Environmental investigation and monitoring	o3	1.09
11	Identification & Reconnaissance	o2	1.09	23	Environmental remediation	e1	1.09
12	Division of the alert area	e3	1.09	24	Information distribution	e2	1.09

**Table 9:** Human error probability adjustment values.

No.	Emergency Activities	Error Probability Basic Values	Weighting Factors	Error Probability Adjustment Values
1	Initial handling of the accident	0.03	1.09	0.0327
2	Reporting of accident information	0.003	1.09	0.00327
3	Acceptance of accident information	0.0005	1.09	0.000545
4	Notice to emergency teams	0.003	1.09	0.00327
5	Collaborative linkage unit	0.01	1.06	0.0106
6	Mobilization of emergency resources	0.01	1.06	0.0106
7	Emergency command	0.01	1.06	0.0106
8	Evacuation & Isolation	0.0005	1.09	0.000545
9	Maintenance of traffic order	0.003	1.09	0.00327
10	Inquiries	0.003	1.09	0.00327
11	Identification & Reconnaissance	0.007	1.09	0.00763
12	Division of the alert area	0.0005	1.09	0.000545
13	Risk control	0.003	1.09	0.00327
14	Safety protection	0.003	1.09	0.00327
15	Personnel rescue	0.003	1.09	0.00327
16	Disposal of leaked substances	0.003	1.09	0.00327
17	Disposal of leaked source	0.003	1.09	0.00327
18	Disposal of transport vehicles	0.003	1.09	0.00327
19	Information feedback	0.003	1.09	0.00327
20	Cleanup of leakage site	0.03	1.09	0.0327
21	Decontamination	0.003	1.09	0.00327
22	Environmental investigation and monitoring	0.007	1.09	0.00763
23	Environmental remediation	0.003	1.09	0.00327
24	Information distribution	0.003	1.09	0.00327

Note: Error probability adjustment values = Failure probability basic values × Weighting factors in Table 9.

**Table 10:** Human error level classification.

Level Classification	Emergency Activities	Number
High	initial handling of the accident, cleanup of leakage site	2
Middle	collaborative linkage unit, mobilization of emergency resources, emergency command, collaborative linkage unit, mobilization of emergency resources, emergency command	5
Low	reporting of accident information, notice to emergency teams, maintenance of traffic order, inquiries, risk control, safety protection, personnel rescue, disposal of leaked substances, disposal of leaked source, disposal of transport vehicles, information feedback, decontamination, environmental remediation, information distribution, accident information, evacuation and isolation, division of the alert area	17

## References

[1] Wu W., Lin J. (2015). 100 Cases of Transportation Accident Involving Hazardous Chemical Materials in China for Last Decade. Logist. Technol..

[2] Xia Y. (2015). Research on Emergency Decision-Making for Unconventional Disasters and Accidents Based on Scenario-Response.

[3] Berman O., Verter V., Kara B.Y. (2007). Designing emergency response networks for hazardous materials transportation. Comput. Oper. Res..

[4] Jabbari M., Atabi F., Ghorbani R. (2020). Key airborne concentrations of chemicals for emergency response planning in HAZMAT road transportation- margin of safety or survival. J. Loss Prev. Process Ind..

[5] Chen W., Shi Y. (2004). Study of Emergency Rescue System of Chemical Accident in Road Transportation. China Saf. Sci. J..

[6] Yu Q., Jiang J., Yu H. (2015). Study on Graphic Demonstration Technology for Emergency Decision-making in Event of Highway Accident Involving Hazardous Chemical Materials. Logist. Technol..

[7] Fabiano B., Curro’ F., Reverberi A., Pastorino R. (2005). Dangerous good transportation by road: From risk analysis to emergency planning. J. Loss Prev. Process Ind..

[8] Zhang C., Chen X., Chen J., Liu Y. (2009). Risk Assessment of Dangerous Chemical Leakage with Emergency Response. J. Tsinghua Univ. (Sci. Technol.).

[9] Turoff M., Chumer M., Walle B.V. (2004). The Design of a Dynamic Emergency Response Management Information System (DERMIS). J. Inf. Technol. Theory Appl..

[10] Wang W. (2003). The Research of Emergency Information System of Ships Pollution Accidentally in the Pearl River Estuary Based on MapOjbects.

[11] Wang J., Ruan Z., Zhao Y. Analysis on the Current Situation of Emergency Rescue and Disposal of Hazardous Chemicals Road Transportation Accidents. Proceedings of the 2015 Annual Science and Technology Conference of China Fire Protection Association.

[12] Reason J.T. (1990). Human Error.

[13] Shappell S.A., Wiegmann D.A. (2000). The Human Factors Analysis and Classification System-HFACS. Am. Libr..

[14] Fu G., Lu B., Chen X. (2005). Behavior Based Model for Organizational Safety Management. China Saf. Sci. J..

[15] Hollnagel E. (1998). Cognitive Reliability and Error Analysis Method: CREAM.

[16] Calhoun J., Savoie C., Randolph-Gips M., Bozkurt I. (2012). Human Reliability Analysis in Spaceflight Applications. Qual. Reliab. Eng. Int..

[17] Lee S.M., Ha J.S., Seong P.H. (2011). CREAM-based communication error analysis method (CEAM) for nuclear power plant operators’ communication. J. Loss Prev. Process Ind..

[18] Huang B., Chen Z. (2011). Types and Causes of Human Error in Take off Phase of Aircraft. Chin. J. Ergon..

[19] Fu Q., Chen Y., Deng Q. (2011). Practical Research into the Cognitive Reliability and Error Analysis of the Human Factors in Traffic Accidents. J. Saf. Environ..

[20] Marseguerra M., Zio E., Librizzi M. (2006). Quantitative developments in the cognitive reliability and error analysis method (CREAM) for the assessment of human performance. Ann. Nucl. Energy.

[21] Felice F.D., Petrillo A., Zomparelli F. (2016). A Hybrid Model for Human Error Probability Analysis. IFAC Pap..

[22] Chai S., Yu J., Du Z., Jing W., Zhou Q. (2011). Quantitative Human Reliability Analysis Methods and Application of Offshore Engineering. J. Tianjin Univ..

[23] Snorre S. (2002). Methods for Accident Investigation.

[24] Sothivanan S. (2015). Laconic Study on Incident/Accident Investigation Technique-Sequentially Timed Event Plotting (STEP). Int. J. Sci. Res. Dev..

[25] Zhang G., Wang Y., Zhang T. (2016). Sequentially Timed Events Plotting in the Application of the Petrochemical Accident Analysis. Saf. Health Environ..

[26] Kang Y. Research on Scheduling Information Model Based on Event Sequence Chain. Proceedings of the International Conference on Advanced Power System Automation and Protection.

[27] Herrera I., Woltjer R. (2010). Comparing a multi-linear (STEP) and systemic (FRAM) method for accident analysis. Reliab. Eng. Syst. Saf..

[28] Kontogiannis T., Leopoulos V., Marmaras N. (2000). A comparison of accident analysis techniques for safety-critical man–machine systems. Int. J. Ind. Ergon..

[29] (2019). Ministry of Emergency Management of the People’s Republic of China. https://www.mem.gov.cn/gk/sgcc/tbzdsgdcbg/.

[30] China Chemical Safety Association (2021). Accident Cases. https://www.chemicalsafety.org.cn/channel/jm8v962jv92y4d1k.

[31] (2021). Ministry of Emergency Management Chemical Registration Center, Chemical Accident Information Network. https://accident.nrcc.com.cn:9090/Portalsite/Index.aspx.

